# Innovating Communication With Social Ties: Diversity in Older Adult Information and Communication Technology Use

**DOI:** 10.1093/geroni/igaf122.1345

**Published:** 2025-12-31

**Authors:** Jess Francis-Levin, Toni Antonucci

## Abstract

When it comes to understanding information and communication technology (ICT) use among older adults, context matters. This symposium will explore such varied contexts with diverse methodologies and samples all centered around the theme of ICT-facilitated social engagement among older adults. First, Suh and colleagues analyzed the Health and Retirement Study and identified four distinct ICT user profiles; they found that user profile membership served as a moderator between social support and future wellbeing. Next, Francis-Levin and Dogari, employ a mixed-methods approach to explore the motivations for social media use among LGBTQ+ older adults (55+) and younger (18-54) adults. Their findings indicate a difference in motivation as older adults are significantly more motivated than younger adults to use social media for social bonding and in responses to family request. Qualitative findings will also be discussed. Third, Ajrouch and Francis-Levin will present findings from their online survey of older Facebook users (65+) who live alone. Findings from their analysis of the different modes of Facebook communication and the impact on perceived social support indicate a significant and positive association with the frequency of direct communication on Facebook and perceived social support. Finally, Choi and colleagues will present their findings from a scoping review on the use of technology for access to social programs and care during the Covid-19 pandemic among older adults with cognitive impairments. Their findings illuminate benefits and challenges, as well as contextual factors impacted by the increased exposure to technology use to access both social and health support.

